# Appearance-related distress impacts psychological symptoms in Chinese patients with cleft lip

**DOI:** 10.3389/fpubh.2025.1484025

**Published:** 2025-01-23

**Authors:** Yichun Yang, Renjie Yang, Jiaying Wang, Zhuojun Xie, Yuan Zong, Weiyao Xia, Meijun Du, Shiming Zhang, Jiayi Yin, Jiali Chen, Bing Shi, Caixia Gong, Hanyao Huang

**Affiliations:** ^1^State Key Laboratory of Oral Diseases and National Clinical Research Center for Oral Diseases and Department of Oral and Maxillofacial Surgery, West China Hospital of Stomatology, Sichuan University, Chengdu, Sichuan, China; ^2^State Key Laboratory of Oral Diseases and National Clinical Research Center for Oral Diseases and Eastern Clinic, West China Hospital of Stomatology, Sichuan University, Chengdu, Sichuan, China

**Keywords:** cleft lip, mental health, anxiety, depression, quality of life

## Abstract

**Background:**

This study aimed to explore the characteristics of appearance-related distress and the relationship between appearance-related distress, anxiety and depression symptoms in Chinese patients with cleft lip (CL).

**Methods:**

The Derriford Appearance Scale 59 (DAS-59), Generalized Anxiety Disorder-7 (GAD-7), and Patient Health Questionnaire-9 (PHQ-9) were used to screen for appearance-related distress, anxiety, and depression symptoms in Chinese patients with CL, respectively.

**Results:**

A total of 63 patients with CL, comprising 43 unilateral and 20 bilateral cases, and 64 individuals without CL in the control group were included in the study. Appearance-related distress was compared between patients with CL and non-cleft individuals. The relationship between appearance-related distress and two psychological symptoms was estimated. The DAS-59 scores in patients with CL were significantly higher than those in non-cleft individuals. The DAS-59 scores in patients with CL who had anxiety or depression symptoms were significantly higher than those without symptoms, and the DAS-59 scores were correlated with GAD-7 and PHQ-9 scores. After adjustment for demographic variables, the DAS-59 scores were also positively associated with GAD-7 and PHQ-9 scores in patients with CL.

**Conclusion:**

More significant appearance-related distress was observed in Chinese patients with CL compared to the control group, but it did not exhibit a correlation with the patients’ diagnosis (unilateral or bilateral), sex, age, or other demographic characteristics. Furthermore, appearance-related distress plays a significant role in psychological symptoms and may serve as a predictor of anxiety and depressive symptoms.

## Introduction

Cleft lip (CL) needs surgical repair at a very young age, but with growth, secondary CL and nasal differences can occur (1). Despite current surgical techniques endeavoring to restore the perfect shape of the nasolabial structure with minimal facial scarring, appearance in patients with CL is rarely perfect, and residual facial differences can still be noticed.

Patients with CL often experience discrimination and negative judgments from society and their peers due to their noticeable facial differences (2–4). Such social discrimination can result in heightened self-consciousness and feelings of shame, thereby increasing their psychological burden. Research indicates that appearance-related distress is common among patients with CL. Prolonged social exclusion and negative feedback can contribute to feelings of inferiority and shame, significantly compromising their overall quality of life and mental well-being (5–7).

Research indicates that anxiety and depression symptoms are generally prevalent among CL patients. Approximately 28–44% of CL patients experience anxiety, while 40–55% suffer from depression (2, 8–10). A cross-sectional study found that 80% of patients with CL experienced at least one psychological issue, a significantly higher proportion compared to the non-CL population (9). The symptoms of anxiety and depression have a significant impact on the quality of life of these patients, making it difficult for them to participate in social activities, maintain interpersonal relationships, and achieve personal goals. This results in decreased quality of life, increased psychological burden, and difficulties in social adaptation. Understanding the relationship between appearance-related distress and the symptoms of anxiety and depression is essential for developing effective psychological interventions and treatment strategies. Thorough research into this relationship can facilitate the creation of more personalized and targeted interventions, helping patients improve their self-esteem and coping skills, thereby enhancing their psychological health.

Although prior studies have examined the impact of appearance-related distress on mental health, the specific relationship between appearance-related distress and symptoms of anxiety and depression in patients with CL within the Chinese cultural context has not been adequately explored. To the best of our knowledge, this study is the first to use the validated Chinese version of the Derriford Appearance Scale 59 (DAS-59) to collect data from the CL population, including minors. Our work might provide a foundation for developing of more tailored psychological interventions and support systems in the future.

Based on the above theoretical framework, this study proposes the following hypotheses: (1) Individuals with CL exhibit higher levels of appearance-related distress compared to those without CL; (2) Within group of the patient with CL, there is a positive correlation between appearance-related distress and symptoms of anxiety and depression.

## Materials and methods

### Study subjects

The study group consisted of patients with CL meeting the inclusion and exclusion criteria and who visited West China Hospital of Stomatology, Sichuan University from July 2019 to October 2019. The inclusion criteria of the study group were: (i) no syndromic CL; (ii) underwent CL repairing; (iii) aged over 10 years; and (iv) able to comprehend and fill out the questionnaires. The researchers initially explained the purpose and procedures of the study to the participants or their legal guardians (in the case of minors) and obtained written informed consent before participation. For adult participants, the researchers distributed paper-based questionnaires and did not provide any additional information unless participants had specific inquiries regarding the questionnaire. For minors, the researchers collaborated with the participants’ guardians to facilitate the participants’ understanding and completion of the questionnaire. They assisted in clarifying any ambiguous or unclear content. All study procedures were completed before the patients underwent surgery to ensure that the effects of surgical trauma would not confound the questionnaire responses.

The control group was recruited using convenience sampling from the same geographic area and during the same period as the study group, with participants sourced from the Hongmen Street Community in Sichuan Province, China. The inclusion criteria of the control group were: (i) without maxillofacial differences or other critical diseases; (ii) no history of esthetic surgery; (iii) aged over 10 years; and (iv) able to comprehend and fill out the questionnaires. Similar to the study group, control group participants were also required to complete the paper-based questionnaire one-on-one with the assistance of the researchers. This approach ensured consistency in the data collection process between the two groups. Given that all questionnaires were completed with the researchers’ one-on-one assistance, this contributed to the accuracy and reliability of the data collected.

In total, 63 patients with CL, including 43 with unilateral CL and 20 with bilateral CL, and 64 individuals in the control group were included in this study and successfully completed all questionnaires.

### Questionnaires

#### Sociodemographic data

Sociodemographic information of participants was collected for subsequent analyses, including sex (male, female), age, region (urban, rural), family income (low: monthly family income ≤3,000 yuan; high: monthly family income >3,000 yuan) and educational level (incomplete compulsory school, finished compulsory school and higher).

#### DAS-59

The DAS-59 is a widely used instrument for assessing psychological distress and dysfunction in individuals with appearance concerns. The dysfunction described refers to physical limitations resulting from psychological distress, such as avoidance behaviors and social withdrawal (11). The Chinese version of this instrument has been proven a valuable tool with sustainable reliability and validity (12). The Cronbach’s alpha coefficient for the DAS-59 full scale is 0.951, reflecting the high reliability of the DAS-59 used in this study (12). The DAS-59 consists of 59 items, each rated on a five-point Likert scale, where participants rate the frequency of symptoms (1 = almost never, 5 = almost always) and the level of emotional distress (1 = not at all distressed, 5 = extremely distressed). The scale is divided into five subscales that measure different aspects of appearance-related psychological discomfort, including: General self-consciousness of appearance (GSC), Social self-consciousness of appearance (SSC); Sexual and body self-consciousness of appearance (SBSC); Negative self-concept (NSC); and Facial self-consciousness of appearance (FSC). The NSC scale is an inverted scale. To compute the NSC score, the original responses are reversed (i.e., 6 minus the original score). This reversed score is then combined with the scores from the other subscales to obtain the overall DAS-59 score. To maintain consistency with existing research, NSC scores are presented in the results as original scores, meaning that higher scores reflect less negative self-evaluation and a more positive overall self-concept.

The total DAS-59 score is calculated by summing the scores from all items. Higher scores indicate a greater severity of distress and dysfunction.

#### Generalized Anxiety Disorder-7 (GAD-7)

The scale is a 7-item self-report tool for screening generalized anxiety and evaluating the severity of anxiety symptoms (13, 14). The Chinese version of GAD-7 is a valid instrument for measuring subjects’ anxiety-related symptoms over the last 2 weeks, with good reliability and validity and calculated Cronbach’s alpha coefficient for 0.928 (15, 16). There are seven items on this scale, with the option of 0 = never, 1 = a few days, 2 = more than half a week, and 3 = almost every day. The total score is achieved by summing up all the seven items and cut-off scores of 5, 10, and 15, represent mild, moderate, and severe anxiety, accordingly. The GAD-7 score of more than 5 points is considered as indicating anxiety symptoms in this study (17).

#### Patient Health Questionnaire-9 (PHQ-9)

The questionnaire is designed to screen and evaluate depression symptoms (18, 19). The Chinese version of PHQ-9 is an effective tool for measuring participants’ depression-related symptoms over the last 2 weeks, with good reliability and validity (20). It consists of nine items evaluated on a Likert scale ranging from 0 to 3 (0 = none at all.... 3 = nearly every day). A summed score is obtained by adding the scores of all nine items and dividing it into four levels: no symptoms (0–4), mild (5–9), moderate (10–14), moderately severe (15–19), and severe (20–27). The PHQ-9 score of more than 5 points is considered as indicating depression symptoms in this study (19). The Cronbach’s alpha coefficient for the PHQ-9 is 0.860, indicating high reliability and internal consistency (20).

### Statistical analysis

All statistics analyses were conducted using SPSS 24.0. All of the data were analyzed using two-sided tests, and *p*-value <0.05 was considered statistically significant.

G*Power 3.1 was used for the sample size calculation. We determined that the correlation coefficient 𝑟 between the DAS-59 score and the GAD-7 or PHQ-9 scores needs to exceed 0.4 to indicate a clinically significant correlation. Therefore, assuming a correlation 𝜌 under the alternative hypothesis (H1) of 0.4, a correlation 𝜌 under the null hypothesis (H0) of 0, with an *α* level set at 0.05 and a power (1-*β*) of 0.8, the total sample size calculated using the following formula is 47.

$$N={\left(\frac{Z_{\alpha /2}+Z_{\beta }}{\mathrm{arctanh}\left(\rho_{H1}\right)-\mathrm{arctanh}\left(\rho_{H0}\right)}\;\right)}^{2}+3$$

$$\mathrm{arctanh}\left(\rho \right)=\frac{1}{2}\ln\left(\frac{1+\rho }{1-\rho }\right)$$

The demographic characteristics of participants were shown with descriptive analysis. Continuous data were expressed as mean ± standard deviation (SD), and categorical data were expressed as frequency and percentage. Differences in DAS-59 scores between groups were compared by adopting the Wilcoxon rank-sum test. The DAS-59 scores of the study group were compared by the Wilcoxon rank-sum test, while demographic characteristics were used as the grouping variables. Differences in DAS-59 scores between patients with and without psychological symptoms were examined by the Wilcoxon rank-sum test. Spearman correlation analysis was used to correlate DAS-59 scores with GAD-7 scores and PHQ-9 scores. The degree of correlation was classified as follows: weak correlation, r < 0.30; moderate correlation, 0.30 ≤ r ≤ 0.50; and strong correlation, r > 0.50. Based on correlation analysis, a multivariate linear regression test was used to determine predictors of GAD-7 and PHQ-9 scores, adjusted for the demographic characteristics of the study group. We also performed tests for multicollinearity by assessing the variance inflation factor (VIF) for each independent variable within our multivariate linear regression models. The *p*-values of Wilcoxon rank-sum test, Spearman correlation analysis and multivariate linear regression test, were adjusted with Benjamini and Hochberg method.

## Results

### Descriptive data

Ultimately, there were 127 participants who completed the questionnaires. There were 63 participants in the study group (mean age: 17.10 ± 4.65 years) and 64 in the control group (mean age: 17.30 ± 3.32 years). The demographic characteristics of the study and control groups are shown in Table 1.

**Table 1:** The demographic characteristics in the study group and control group.

Variables		Study group	Control group
Sex	Male	43 (68%)	27 (42%)
	Female	20 (32%)	37 (58%)
Age	Mean ± SD	17.10 ± 4.65	17.30 ± 3.32
	Median (IQR)	18 (14, 20)	18 (14, 19)
Region	Urban	22 (35%)	20 (31%)
	Rural	41 (65%)	44 (69%)
Family income	Low	37 (69%)	14 (22%)
	High	26 (31%)	50 (78%)
Educational level	Incomplete compulsory school	30 (48%)	27 (42%)
	Finished compulsory school and higher	33 (52%)	37 (58%)
Diagnosis	Unilateral CL	43 (68%)	–
	Bilateral CL	20 (32%)	–

Data were shown as *N* (%). CL, cleft lip; SD, standard deviation; IQR, interquartile range.

### The DAS-59 scores of the study and control groups

The DAS-59 scores of the study and control groups were compared. The full-scale score, GSC score and SSC score in the study group were significantly higher than those of the control group, NSC score was significantly lower than that of the control group (Table 2).

**Table 2:** Differences of DAS-59 scores between the study group and control group.

Domains	Study group (*N* = 63)	Control group (*N* = 64)	*B*	*p* value
Full scale	137.44 ± 33.37	123.30 ± 26.12	16.46	0.002*
GSC	43.95 ± 11.86	38.81 ± 10.77	5.86	0.003*
SSC	42.64 ± 13.76	33.58 ± 10.16	9.50	0.002*
SBSC	19.68 ± 6.10	18.08 ± 5.30	2.57	0.013*
NSC	14.95 ± 4.13	18.16 ± 3.64	−3.02	0.002*
FSC	7.73 ± 3.37	7.83 ± 2.62	−0.13	0.825

*p < 0.05 by multivariate linear regression test, adjusted for sex, age, region, family income and educational level. The *p*-values were corrected using the Benjamini and Hochberg method. GSC, General self-consciousness; SSC, Social self-consciousness; NSC, Negative self-concept; SBSC, Sexual and bodily self-consciousness; FSC, Facial self-consciousness. Bold indicates significant values.’

### Comparison of DAS-59 scores by sociodemographic variables

Diagnosis, sex, age, region, family income, and educational level were used as grouping variables to compare the differences in DAS-59 scores between the study group using the Wilcoxon rank-sum test. There were no significant differences related to all the above grouping variables (Supplementary Table 1).

### GAD-7 and PHQ-9 scores of Chinese patients with CL

The mean GAD-7 and PHQ-9 scores in the study group were 4.175 ± 3.387 and 5.444 ± 4.717, respectively (Supplementary Tables 2, 3). The majority of study group participants were without anxiety and depression symptoms. There were 20 study group participants (31.7%) with anxiety symptoms as well as 30 study group participants (47.6%) with depression symptoms in those with CL.

### Comparison of DAS-59 scores between study group participants with or without psychological symptoms

The full scale (154.95 ± 33.87), GSC (51.15 ± 12.18), SSC (49.25 ± 13.91), and SBSC (22.80 ± 6.77) scores of DAS-59 in study group with anxiety symptoms were significantly higher than those without anxiety symptoms, while the NSC (13.45 ± 3.40) score was lower. Additionally, the full scale (149.73 ± 33.61), GSC (49.20 ± 11.83) and SSC (47.93 ± 13.10) scores of DAS-59 in study group with depression symptoms were higher than those without depression symptoms, and the differences were statistically significant (Table 3). Subsequently, we assessed the differences in DAS-59 scores between participants exhibiting both anxiety and depression symptoms and those without any emotional symptoms. It showed that participants in study group with both anxiety and depression symptoms had significantly higher scores on the DAS-59 full scale, GSC, SSC, and SBSC compared to those without any emotional symptoms (*p* < 0.05) (Supplementary Table 4).

**Table 3:** Differences in DAS-59 scores between study group participants with and without psychological symptoms.

Domains	With or without anxiety symptoms		*p* value
	Without anxiety (*N* = 43)	With anxiety (*N* = 20)	
Full scale	129.30 ± 30.19	154.95 ± 33.87	0.012*
GSC	40.61 ± 10.22	51.15 ± 12.18	0.012*
SSC	39.56 ± 12.85	49.25 ± 13.91	0.012*
SBSC	18.24 ± 12.70	22.80 ± 6.77	0.012*
NSC	15.65 ± 4.28	13.45 ± 3.40	0.022*
FSC	7.33 ± 3.34	8.60 ± 3.35	0.117

Domains	With or without depression symptoms		*p* value
	Without depression (*N* = 33)	With depression (*N* = 30)	
Full scale	126.27 ± 29.39	149.73 ± 33.61	0.010*
GSC	39.18 ± 9.82	49.20 ± 11.83	0.006*
SSC	37.82 ± 12.68	47.93 ± 13.10	0.006*
SBSC	18.42 ± 5.57	21.07 ± 6.44	0.129
NSC	15.73 ± 4.61	14.10 ± 3.40	0.143
FSC	7.58 ± 3.35	7.90 ± 3.44	0.706

*p < 0.05 by Wilcoxon rank-sum test. The *p*-values were corrected using the Benjamini and Hochberg method. Bold indicates significant values.’

### The correlation of DAS-59 scores with GAD-7 and PHQ-9 scores

In the study group, the GAD-7 score was moderately correlated with full scale score (*r* = 0.485, *p* < 0.001), SSC score (*r* = 0.481, *p* < 0.001) and SBSC score (*r* = 0.469, *p* < 0.001), and strongly correlated with the GSC score (*r* = 0.510, *p* < 0.001). The PHQ-9 score was moderately correlated with full scale score (*r* = 0.403, *p* = 0.002), GSC score (*r* = 0.416, *p* = 0.002), SSC score (r = 0.490, *p* < 0.001), and SBSC score (*r* = 0.350, *p* = 0.007), and weakly correlated with the NSC score (*r* = −0.271, *p* = 0.038) (Table 4).

**Table 4:** Correlations between GAD-7, PHQ-9 scores and DAS-59 scores.

Domains	GAD-7		PHQ-9	
	*r*	*p* value	*r*	*p* value
Full scale	0.485*	**<0.001**	0.403*	**0.002**
GSC	0.510*	**<0.001**	0.416*	**0.002**
SSC	0.481*	**<0.001**	0.490*	**<0.001**
SBSC	0.469*	**<0.001**	0.350*	**0.007**
NSC	−0.299*	**0.021**	−0.271*	**0.038**
FSC	0.248	0.050	0.126	0.327

*p < 0.05 by Spearman correlation analysis. The *p*-values were corrected using the Benjamini and Hochberg method. Bold indicates significant values.’

The non-adjusted model showed that full scale score, GSC score, SSC score, and SBSC score were positively related to the GAD-7 score in multivariate linear regression analysis. After adjusting for diagnosis, sex, age, region, family income and educational level, the association between the DAS-59 scores and the GAD-7 score was still significant. For each 1-point increase in the full scale score, GSC score, SSC score, and SBSC score, the GAD-7 score increased by 0.056 (95% CI = 0.033 ~ 0.078), 0.165 (95% CI = 0.102 ~ 0.229), 0.127 (95% CI = 0.071 ~ 0.183), and 0.309 (95% CI = 0.184 ~ 0.434), respectively (Table 5).

**Table 5:** Multivariate linear regression analysis between the DAS-59 scores and the GAD-7 scores and the PHQ-9 scores, adjusted for diagnosis, sex, age, region, family income and educational level.

Domains	Non-adjusted model 1		Adjusted model 1		Non-adjusted model 2		Adjusted model 2	
	B (95%CI)	*p*	B (95%CI)	*p*	B (95%CI)	*p*	B (95%CI)	*p*
Full scale	0.055 (0.034 ~ 0.077)	<0.001*	0.056 (0.033 ~ 0.078)	<0.001*	0.055 (0.034 ~ 0.077)	<0.001*	0.077 (0.045 ~ 0.109)	<0.001*
GSC	0.155 (0.093 ~ 0.216)	<0.001*	0.165 (0.102 ~ 0.229)	<0.001*	0.217 (0.132 ~ 0.302)	<0.001*	0.227 (0.136 ~ 0.317)	<0.001*
SSC	0.125 (0.070 ~ 0.179)	<0.001*	0.127 (0.071 ~ 0.183)	<0.001*	0.184 (0.110 ~ 0.258)	<0.001*	0.194 (0.117 ~ 0.270)	<0.001*
SBSC	0.309 (0.191 ~ 0.427)	<0.001*	0.309 (0.184 ~ 0.434)	<0.001*	0.363 (0.188 ~ 0.538)	<0.001*	0.379 (0.194 ~ 0.565)	<0.001*
NSC	−0.191 (−0.395 ~ 0.013)	0.067	−0.189 (−0.413 ~ 0.036)	0.098	−0.263 (−0.548 ~ 0.022)	0.084	−0.255 (−0.571 ~ 0.061)	0.112
FSC	0.308 (−0.063 ~ 0.553)	0.018*	0.318 (−0.047 ~ 0.588)	0.026*	0.286 (−0.065 ~ 0.637)	0.108	0.321 (−0.069 ~ 0.710)	0.112

*p < 0.05 by multivariate linear regression test. The *p*-values were corrected using the Benjamini and Hochberg method. Model 1: Multivariate linear regression analysis between the DAS-59 scores and the GAD-7 scores. Model 2: Multivariate linear regression analysis between the DAS-59 scores and the PHQ-9 scores. Bold indicates significant values.’

**Figure 1:**
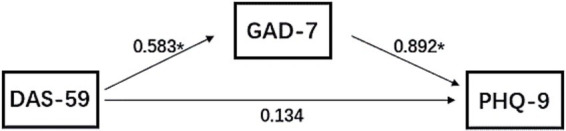
Structural equation model of the relationship between DAS-59, GAD-7, and PHQ 9. The numbers on the arrows indicate the path coefficients, *p < 0.001.

In further analysis, we developed a structural equation model to examine the relationships among DAS-59, PHQ-9, and GAD-7 (Figure 1). We found that DAS-59 completely mediated the impact on PHQ-9 through GAD-7 (standardized effect = 0.583, *p* < 0.001), without exerting a direct effect. The standardized effect of GAD-7 on PHQ-9 was 0.892 (*p* < 0.001).

## Discussion

This study aimed to explore the relationship between appearance-related distress and symptoms of anxiety and depression among patients with CL. Compared to those without anxiety or depression, patients with CL with psychological symptoms exhibit significantly higher appearance-related distress. Furthermore, nearly all domains of the DAS-59 are significantly correlated with psychological symptoms, indicating that greater severity of appearance-related distress is associated with more pronounced states of anxiety and depression in patients.

Given that surgeon-reported outcomes may not accurately match patients’ expectations for repair outcomes (5), the subjective self-assessment, as reported by the patient, has become increasingly valuable in health care evaluation (21). In patients with CL, the self-consciousness of their appearance may arise with the growth and distress the patients when the facial morphology is different, and then affect the patient’s psychological symptoms. This can then affect their psychological symptoms. This study first attempted to measure appearance-related distress among patients with CL using the DAS-59. We found that patients with CL experienced more distress than patients without CL. The study group showed higher scores in the GSC subscale and lower scores in the NSC subscale compared to non-cleft controls. It was evident that cleft characteristics affected the global self-awareness of appearance and positive self-acceptance in Chinese patients with CL, consistent with previous studies (22–24). Higher scores in the SSC domain suggested that social interactions could be challenging for patients with CL. This could be because those who look different are more likely to experience staring, comments, and questions about their appearance, which could interfere with their routine social interactions (25). There were no significant differences in the SBSC subscale, as injury and physical dysfunction associated with appearance may not be main concerns for patients with CL (24). The lack of prominent difference in the FSC subscale may result from the constraint of using a general questionnaire containing items not specifically targeted to CL groups (such as “Distress from being unable to change hairstyle” and “Avoid getting the hair wet”). Our study indicated that after CL repair, patients with CL were still dissatisfied with their appearance. Thus, patients seemed to be more concerned with visible abnormalities than functional recovery.

We found that the degree of appearance-related distress does not appear to be significantly influenced by socio-demographic factors. The effect size in the adjusted model did not change significantly, which further supports this conclusion. This suggests that appearance-related distress in patients with CL may be primarily shaped by individual self-perception rather than socio-demographic factors. Additionally, given that this study employed a single-center design, it is possible that these variables were evenly distributed within the sample, which may account for their lack of significant effects to some extent. Finally, this result may indicate the widespread nature of appearance-related distress across populations, suggesting that cross-cultural and socio-contextual factors warrant deeper exploration in future research.

Our study demonstrated that Chinese patients with CL with anxiety or depression symptoms were more concerned about their appearance than those without the two psychological symptoms. Spearman correlation analysis and multivariate linear regression test also proved that appearance-related distress was a significant predictor of anxiety and depression symptoms. It is consistent with previous studies in which correlations between emotional well-being and satisfaction with general appearance had been confirmed in patients with CL (6, 26, 27). Patients with CL are more likely to be worried about social acceptance on account of apprehension about facial appearance (27). Meanwhile, the visibility of a cleft had a prominent and passive influence on intimate relationships (28). That may partially explain the results, and the relevance of SSC subscale score with GAD-7 and PHQ-9 scores also proved that. Therefore, it may be possible to identify individuals at risk of psychological problems by measuring appearance-related distress.

This study discovered a significant positive correlation between appearance-related distress and anxiety symptoms in CL patients. Furthermore, anxiety symptoms, acting as a mediating factor, may lead to the development of depressive symptoms. These findings indicate that anxiety serves as a crucial intermediary between appearance-related distress and depression. Previous research has indicated a frequent co-occurrence between anxiety and depression, showing a significant positive correlation (29, 30). Moreover, longitudinal follow-up data also demonstrate that the presence of anxiety disorders significantly increases the risk of developing depression, with anxiety often acting as a significant precursor to depression (31, 32).

Anxiety is often triggered by external stressors. For CL patients, concerns about social discrimination and negative evaluations due to appearance-related issues are immediate factors that trigger anxiety. In contrast, depression is usually a more prolonged emotional state that develops over time as negative experiences accumulate (33, 34). This phenomenon has been observed particularly in adolescents, who were the primary focus of this study (35, 36). However, to our knowledge, there is currently a lack of longitudinal studies explicitly indicating whether anxiety significantly triggers depression in CL patients. Therefore, further investigation is needed to understand their relationship.

Our study provides preliminary evidence suggesting that the psychological development of anxiety and depression in the CL group may be comparable to that observed in the non-CL population. The experience of appearance-related distress directly triggers anxiety, and persistent anxiety symptoms can subsequently contribute to the onset of depression. This discovery underscores the importance of effectively assisting CL patients in managing appearance-related anxiety to prevent and alleviate long-term depression.

Cognitive Behavioral Therapy (CBT) has been shown to be effective in treating anxiety disorders by improving emotional regulation and social skills (37). Limited research has investigated its effectiveness in CL patients with anxiety, and CBT has not been extensively implemented in the treatment of CL patients. This study found that appearance-related distress significantly influences psychological distress in patients with cleft lip and may also worsen depressive symptoms through anxiety. These results highlight the important role of appearance-related distress in the psychological health of individuals with cleft lip, suggesting that interventions focusing solely on anxiety and depression may be inadequate in addressing the comprehensive needs of these patients. Therefore, traditional CBT can be further enhanced within the existing framework by incorporating additional components that emphasize appearance cognition, body image, and social acceptance. For example, integrating appearance acceptance training into the therapeutic process could facilitate improvements in patients’ self-image and self-acceptance, thereby reducing anxiety associated with appearance differences. Importantly, when designing personalized interventions, it is essential to account for cultural differences. In certain cultural contexts or regions, patients may attribute greater significance to group identity and social acceptance, which could intensify their psychological distress, especially regarding appearance-related issues. Therefore, interventions should be adjusted according to the patient’s cultural background to better meet their socio-psychological needs, thereby enhancing self-esteem, coping strategies, and overall psychological well-being.

This study is limited by the fact that the subjects came only from one single specialized hospital of stomatology and the sample size of our study can be enhanced in the future. Secondly, although a potential mechanism of appearance-related distress mediating depressive symptoms through anxiety symptoms has been identified, it should be noted that the findings presented in this study are preliminary. To confirm the applicability of this mechanism in patients with CL, future studies with larger sample sizes and longitudinal designs are necessary. Third, although some studies have utilized the DAS-59 in children aged 10 and older, no formal investigations have specifically assessed its sensitivity and specificity within this age group. Psychological responses to appearance-related distress may vary among different age groups of patients with CL. Future research should aim to validate the sensitivity, specificity, and overall performance of the DAS-59 in children and adolescents to better establish its applicability in broader populations. Additionally, age-stratified analyses should be conducted to explore how age affects the psychological impact of appearance distress and to provide a foundation for personalized interventions.

## Conclusion

This study showed that appearance-related distress was present in Chinese patients with CL. More importantly, appearance-related distress is of great importance for psychological symptoms, which could be predictors of anxiety and depression symptoms.

## Data Availability

The raw data supporting the conclusions of this article will be made available by the authors, without undue reservation.

## References

[1] Mossey PA Little J Munger RG Dixon MJ Shaw WC. Cleft lip and palate. Lancet. (2009) 374:1773–85. doi: 10.1016/S0140-6736(09)60695-4, PMID: 19747722

[2] Fadeyibi IO Coker OA Zacchariah MP Fasawe A Ademiluyi SA. Psychosocial effects of cleft lip and palate on Nigerians: the Ikeja-Lagos experience. J Plast Surg Hand Surg. (2012) 46:13–8. doi: 10.3109/2000656X.2011.643027, PMID: 22455571

[3] Guillén AR Peñacoba C Romero M. Psychological variables in children and adolescents with cleft lip and/or palate. J Clin Pediatr Dent. (2020) 44:116–22. doi: 10.17796/1053-4625-44.2.9, PMID: 32271659

[4] Damiano PC Tyler MC Romitti PA Momany ET Jones MP Canady JW. Health-related quality of life among preadolescent children with oral clefts: the mother's perspective. Pediatrics. (2007) 120:e283–90. doi: 10.1542/peds.2006-2091, PMID: 17671039

[5] Mani M Reiser E Andlin-Sobocki A Skoog V Holmström M. Factors related to quality of life and satisfaction with nasal appearance in patients treated for unilateral cleft lip and palate. Cleft Palate Craniofac J. (2013) 50:432–9. doi: 10.1597/11-035, PMID: 22035039

[6] Oosterkamp BCM Dijkstra PU Remmelink HJ van Oort RP Goorhuis-Brouwer SM Sandham A. Satisfaction with treatment outcome in bilateral cleft lip and palate patients. Int J Oral Maxillofac Surg. (2007) 36:890–5. doi: 10.1016/j.ijom.2007.07.008, PMID: 17766083

[7] Hutchinson K Wellman MA Noe DA Kahn A. The psychosocial effects of cleft lip and palate in non-Anglo populations: a cross-cultural meta-analysis. Cleft Palate Craniofac J. (2011) 48:497–508. doi: 10.1597/09-046, PMID: 20815712

[8] Endriga MC Kapp-Simon KA. Psychological issues in craniofacial care: state of the art. Cleft Palate Craniofac J. (1999) 36:3–11. doi: 10.1597/1545-1569_1999_036_0001_piiccs2.3.co_2, PMID: 10067755

[9] Nicholls W Harper C Robinson S Persson M Selvey L. Adult-specific life outcomes of cleft lip and palate in a Western Australian cohort. Cleft Palate Craniofac J. (2018) 55:1419–29. doi: 10.1177/1055665618768540, PMID: 29620916

[10] Piombino P Ruggiero F Dell’Aversana Orabona G Scopelliti D Bianchi A de Simone F. Development and validation of the quality-of-life adolescent cleft questionnaire in patients with cleft lip and palate. J Craniofac Surg. (2014) 25:1757–61. doi: 10.1097/SCS.0000000000001033, PMID: 25010834 PMC4212814

[11] Harris DL Carr AT. The Derriford appearance scale (DAS59): a new psychometric scale for the evaluation of patients with disfigurements and aesthetic problems of appearance. Br J Plast Surg. (2001) 54:216–22. doi: 10.1054/bjps.2001.3559, PMID: 11254413

[12] Sakran KA Song S Li H Pan P Chen N Zeng N. Self-consciousness of appearance in Chinese patients with cleft lip: validation of the Chinese Derriford appearance scale 59 (DAS 59) instrument. Front Pediatr. (2021) 9:825997. doi: 10.3389/fped.2021.825997 35223716 PMC8863654

[13] Spitzer RL Kroenke K Williams JBW Löwe B. A brief measure for assessing generalized anxiety disorder: the GAD-7. Arch Intern Med. (2006) 166:1092–7. doi: 10.1001/archinte.166.10.1092, PMID: 16717171

[14] Mossman SA Luft MJ Schroeder HK Varney ST Fleck DE Barzman DH. The generalized anxiety disorder 7-item scale in adolescents with generalized anxiety disorder: signal detection and validation. Ann Clin Psychiatry. (2017) 29:227–234a. PMID: 29069107 PMC5765270

[15] He XY Li CB Qian J Cui HS Wu WY. Reliability and validity of a generalized anxiety scale in general hospital outpatients. Shanghai Arch Psychiatry. (2010) 22:200–3. doi: 10.3969/j.issn.1002-0829.2010.04.002

[16] Shih YC Chou CC Lu YJ Yu HY. Reliability and validity of the traditional Chinese version of the GAD-7 in Taiwanese patients with epilepsy. J Formos Med Assoc. (2022) 121:2324–30. doi: 10.1016/j.jfma.2022.04.018, PMID: 35584970

[17] Ruiz MA Zamorano E García-Campayo J Pardo A Freire O Rejas J. Validity of the GAD-7 scale as an outcome measure of disability in patients with generalized anxiety disorders in primary care. J Affect Disord. (2011) 128:277–86. doi: 10.1016/j.jad.2010.07.010, PMID: 20692043

[18] Richardson LP McCauley E Grossman DC McCarty CA Richards J Russo JE. Evaluation of the patient health Questionnaire-9 item for detecting major depression among adolescents. Pediatrics. (2010) 126:1117–23. doi: 10.1542/peds.2010-0852, PMID: 21041282 PMC3217785

[19] Kroenke K Spitzer RL Williams JB. The PHQ-9: validity of a brief depression severity measure. J Gen Intern Med. (2001) 16:606–13. doi: 10.1046/j.1525-1497.2001.016009606.x, PMID: 11556941 PMC1495268

[20] Wang W Bian Q Zhao Y Li X Wang W du J. Reliability and validity of the Chinese version of the patient health questionnaire (PHQ-9) in the general population. Gen Hosp Psychiatry. (2014) 36:539–44. doi: 10.1016/j.genhosppsych.2014.05.021, PMID: 25023953

[21] Mani M Carlsson M Marcusson A. Quality of life varies with gender and age among adults treated for unilateral cleft lip and palate. Cleft Palate Craniofac J. (2010) 47:491–8. doi: 10.1597/08-281, PMID: 20180705

[22] Albers AE Reichelt AC Nolst-Trenité GJ Menger DJ. Feeling Normal? Long-term follow-up of patients with a cleft lip-palate after rhinoplasty with the Derriford appearance scale (DAS-59). Facial Plast Surg. (2016) 32:219–24. doi: 10.1055/s-0036-1579781, PMID: 27097144

[23] Kelly SN Shearer J. Appearance and speech satisfaction and their associations with psychosocial difficulties among young people with cleft lip and/or palate. Cleft Palate Craniofac J. (2020) 57:1008–17. doi: 10.1177/1055665620926083, PMID: 32463719 PMC7361652

[24] Ricketts S Regev E Antonyshyn OM Kiss A Fialkov JA. Use of the Derriford appearance scale 59 to assess patient-reported outcomes in secondary cleft surgery. Plastic Surg (Oakville, Ont). (2016) 24:27–31. doi: 10.1177/229255031602400102, PMID: 27054135 PMC4806753

[25] Tiemens K Nicholas D Forrest CR. Living with difference: experiences of adolescent girls with cleft lip and palate. Cleft Palate Craniofac J. (2013) 50:e27–34. doi: 10.1597/10-278, PMID: 22582952

[26] Hunt O Burden D Hepper P Johnston C. The psychosocial effects of cleft lip and palate: a systematic review. Eur J Orthod. (2005) 27:274–85. doi: 10.1093/ejo/cji004, PMID: 15947228

[27] Thomas PT Turner SR Rumsey N Dowell T Sandy JR. Satisfaction with facial appearance among subjects affected by a cleft. Cleft Palate Craniofac J. (1997) 34:226–31. doi: 10.1597/1545-1569_1997_034_0226_swfaas_2.3.co_2, PMID: 9167073

[28] Griffiths C Williamson H Rumsey N. The romantic experiences of adolescents with a visible difference: exploring concerns, protective factors and support needs. J Health Psychol. (2012) 17:1053–64. doi: 10.1177/1359105311433909, PMID: 22253328

[29] Shao R He P Ling B Tan L Xu L Hou Y. Prevalence of depression and anxiety and correlations between depression, anxiety, family functioning, social support and coping styles among Chinese medical students. BMC Psychol. (2020) 8:38. doi: 10.1186/s40359-020-00402-8, PMID: 32321593 PMC7178943

[30] Branson EK Branson VM McGrath R Rausa VC Kilpatrick N Crowe LM. Psychological and peer difficulties of children with cleft lip and/or palate: a systematic review and Meta-analysis. Cleft Palate Craniofac J. (2024) 61:258–70. doi: 10.1177/10556656221125377, PMID: 36082954

[31] Rice F van den Bree MB Thapar A. A population-based study of anxiety as a precursor for depression in childhood and adolescence. BMC Psychiatry. (2004) 4:43. doi: 10.1186/1471-244X-4-43, PMID: 15596007 PMC545489

[32] Avenevoli S Stolar M Li J Dierker L Ries Merikangas K. Comorbidity of depression in children and adolescents: models and evidence from a prospective high-risk family study. Biol Psychiatry. (2001) 49:1071–81. doi: 10.1016/S0006-3223(01)01142-8, PMID: 11430849

[33] Stein MB Sareen J. CLINICAL PRACTICE. Generalized anxiety disorder. N Engl J Med. (2015) 373:2059–68. doi: 10.1056/NEJMcp1502514, PMID: 26580998

[34] Al-Namankany A Alhubaishi A. Effects of cleft lip and palate on children's psychological health: a systematic review. J Taibah Univ Med Sci. (2018) 13:311–8. doi: 10.1016/j.jtumed.2018.04.007, PMID: 31435341 PMC6694901

[35] Reinherz H Stewart-Berghauer G Pakiz B Frost A Moeykens B Holmes W. The relationship of early risk and current mediators to depressive symptomatology in adolescence. J Am Acad Child Adolesc Psychiatry. (1989) 28:942–7. doi: 10.1097/00004583-198911000-00021, PMID: 2808267

[36] Reinherz H Giaconia R Pakiz B Silverman A Frost A Lefkowitz E. Psychosocial risks for major depression in late adolescence: a longitudinal community study. J Am Acad Child Adolesc Psychiatry. (1993) 32:1155–63. doi: 10.1097/00004583-199311000-00007, PMID: 8282659

[37] Kaczkurkin AN Foa EB. Cognitive-behavioral therapy for anxiety disorders: an update on the empirical evidence. Dialogues Clin Neurosci. (2015) 17:337–46. doi: 10.31887/DCNS.2015.17.3/akaczkurkin, PMID: 26487814 PMC4610618

