# Supplementary material for: Appearance-related distress impacts psychological symptoms in Chinese patients with cleft lip
Source: Front Public Health. 2025 Jan 23;13:1484025. doi: 10.3389/fpubh.2025.1484025 (PMC11798753; doi:10.3389/fpubh.2025.1484025)
Supplement: Supplementary file 1 [file Table_1.DOCX]

**Supplemental table 1.** **Factors influencing DAS-59 scores in the study group.**

| Domains | Diagnose | | *P* value |
| --- | --- | --- | --- |
|  | Unilateral cleft lip  (N=43) | Bilateral cleft lip  (N=20) |  |
| Full scale | 136.40±32.70 | 135.84±31.22 | 0.811 |
| GSC | 44.21±12.12 | 43.40±11.56 | 0.811 |
| SSC | 42.35±14.27 | 43.25±12.92 | 0.811 |
| SBSC | 19.00±5.74 | 21.15±6.73 | 0.390 |
| NSC | 15.49±4.03 | 13.80±4.19 | 0.390 |
| FSC | 7.09±3.09 | 9.10±3.61 | 0.390 |
| Domains | Gender | | *P* value |
|  | Female (N=20) | Male (N=43) |  |
| Full scale | 141.25±36.51 | 135.67±32.11 | 0.650 |
| GSC | 45.95±13.52 | 43.02±11.05 | 0.650 |
| SSC | 44.30±16.10 | 41.86±12.66 | 0.650 |
| SBSC | 20.15±6.16 | 19.47±6.14 | 0.682 |
| NSC | 14.30±3.69 | 15.26±4.32 | 0.650 |
| FSC | 8.15±3.07 | 7.54±3.52 | 0.650 |
| Domains | Age | | *P* value |
|  | Nonage (N=41) | Adult (N=22) |  |
| Full scale | 137.88±35.38 | 136.64±30.04 | 0.889 |
| GSC | 43.44±12.82 | 44.91±10.05 | 0.846 |
| SSC | 43.12±14.34 | 41.73±12.87 | 0.846 |
| SBSC | 20.07±6.60 | 18.96±5.10 | 0.846 |
| NSC | 14.51±4.39 | 15.77±3.52 | 0.753 |
| FSC | 8.10±3.62 | 7.05±2.80 | 0.753 |
| Domains | Region | | *P* value |
|  | Urban (N=22) | Rural (N=41) |  |
| Full scale | 134.09±30.05 | 139.18±35.70 | 0.622 |
| GSC | 43.05±10.12 | 44.63±12.90 | 0.622 |
| SSC | 40.73±11.53 | 43.60±15.03 | 0.622 |
| SBSC | 18.59±5.92 | 20.23±6.26 | 0.622 |
| NSC | 15.91±3.18 | 14.33±4.50 | 0.622 |
| FSC | 7.27±3.36 | 7.93±3.42 | 0.622 |
| Domains | Family income | | *P* value |
|  | Low (N=37) | High (N=26) |  |
| Full scale | 141.00±36.55 | 132.39±28.15 | 0.590 |
| GSC | 44.73±13.27 | 42.85±9.65 | 0.647 |
| SSC | 44.32±14.98 | 40.23±11.66 | 0.590 |
| SBSC | 20.11±6.35 | 19.08±5.80 | 0.647 |
| NSC | 14.92±4.62 | 15.00±3.39 | 0.940 |
| FSC | 8.19±3.64 | 7.08±2.88 | 0.590 |
| Domains | Educational level | | *P* value |
|  | Incomplete compulsory school (N=30) | Finished compulsory school and Higher (N=33) |  |
| Full scale | 139.40±37.52 | 135.67±29.58 | 0.661 |
| GSC | 42.40±12.65 | 43.36±11.10 | 0.489 |
| SSC | 44.53±14.93 | 40.91±12.58 | 0.489 |
| SBSC | 20.73±6.83 | 18.73±5.28 | 0.489 |
| NSC | 14.60±4.60 | 15.27±3.69 | 0.626 |
| FSC | 8.40±3.41 | 7.12±3.27 | 0.489 |

Wilcoxon rank-sum test was used. The P-values were corrected using the Benjamini and Hochberg method. GSC: General self-consciousness; SSC: Social self-consciousness; NSC: Negative self-concept; SBSC: Sexual and bodily self-consciousness; FSC: Facial self-consciousness;

**Supplemental table 2.** **GAD-7 score and severity of anxiety in the study group.**

| Mean±SD | Normal,  N (%) | Mild Anxiety,  N (%) | Moderate Anxiety,  N (%) | Severe Anxiety,  N (%) |
| --- | --- | --- | --- | --- |
| 4.18±3.39 | 43 (68.3) | 15 (23.8) | 4 (6.3) | 1 (1.6) |

**Supplemental table 3.** **PHQ-9 score and severity of depression in the study group.**

| Mean±SD | Normal ,  N (%) | Mild Depression,  N (%) | Moderate Depression,  N (%) | Moderately severe Depression, N (%) | Severe Depression,  N (%) |
| --- | --- | --- | --- | --- | --- |
| 5.44±4.72 | 33 (52.4) | 18 (28.6) | 8 (12.7) | 4 (6.3) | 0 (0) |

**Supplemental table 4. Differences in DAS-59 scores between study group participants with and without anxiety and depression symptoms.**

| Domains | Anxiety and depression symptom status | | P value |
| --- | --- | --- | --- |
|  | Without any symptoms (N=29) | With both anxiety and depression (N=16) |  |
| Full scale | 126.97±30.76 | 163.06±32.36 | **0.002*** |
| GSC | 39.14±10.09 | 53.94±11.56 | **0.002*** |
| SSC | 38.24±13.18 | 52.75±12.85 | **0.002*** |
| SBSC | 18.41±5.90 | 24.13±6.73 | **0.012*** |
| NSC | 15.93±4.67 | 13.44±3.37 | 0.080 |
| FSC | 7.59±3.48 | 8.50±3.71 | 0.414 |

**P*<0.05 by Wilcoxon rank-sum test. The P-values were corrected using the Benjamini and Hochberg method.
